# Effect of training status on muscle excitation and neuromuscular fatigue with resistance exercise with and without blood flow restriction in young men

**DOI:** 10.14814/phy2.70274

**Published:** 2025-03-20

**Authors:** Brett H. Davis, Guillaume Spielmann, Neil M. Johannsen, Victoria Fairchild, Timothy D. Allerton, Brian A. Irving

**Affiliations:** ^1^ Louisiana State University Baton Rouge Louisiana USA; ^2^ Pennington Biomedical Research Center Baton Rouge Louisiana USA; ^3^ Present address: University of Tennessee Knoxville Tennessee USA

**Keywords:** blood flow restriction, fatigue, muscle activation, occlusion training, resistance exercise, surface electromyography

## Abstract

This study compared muscle (vastus lateralis) excitation, muscle activation, and neuromuscular fatigue in response to low‐load resistance exercise with blood flow restriction (LLBFR), medium‐load resistance exercise with blood flow restriction (MLBFR), and high‐load resistance exercise (HLRE) in resistance‐trained (RT; *n* = 15) and untrained (UT; *n* = 14) college‐aged males. Muscle excitation and activation were measured using surface electromyography (sEMG) and defined as the maximal root mean square amplitudes (RMS AMP) and the integrated area under the sEMG curve (iEMG) per repetition. Neuromuscular fatigue was defined as the reduction in peak torque measured during the postexercise knee extensor maximal isometric contractions (MVIC) relative to the pre‐exercise MVIC. The LLBFR sessions showed 23.7% (*p* < 0.01) lower relative muscle excitation than the MLBFR and 26.7% (*p* < 0.001) lower than the HLRE. In contrast, LLBFR sessions showed 38.1% (*p* < 0.001) higher total muscle activation than the MLBFR and 19.3% (*p* < 0.05) higher than the HLRE. There were no differences between the RT and UT groups for percent change in peak torque or the RMS AMP measured during the knee extensor MVICs following the three exercise treatments (*p* > 0.05). However, the peak torque and maximal RMS amplitudes were higher in the RT group than in the UT group measured during the pre‐exercise MVICs. Our data suggest that the LLBFR led to greater total muscle activation than MLBFR and HLRE despite lower relative muscle excitation independent of training status in our college‐aged males.

## 1 INTRODUCTION

Resistance exercise (RE) training to task failure, or near‐task failure, is generally considered essential for maximizing skeletal muscle hypertrophy (Morton, Sonne, et al., 2019; Refalo et al., 2022, 2023, 2024). Mechanistically, RE training‐induced muscle hypertrophy is likely enhanced when the number of motor units recruited and firing frequencies are maximized (i.e., greater muscle excitation) (Jenkins et al., 2015; Schoenfeld, 2013; Valerio et al., 2023), particularly when associated with neuromuscular fatigue due to metabolic stress (Schoenfeld, 2013). Moreover, RE training using loads ≥60% of one repetition maximum (1‐RM) or high‐load resistance exercise (HLRE) is important for optimizing skeletal muscle excitation while also enhancing the recruitment of type II muscle fibers (Morton, Sonne, et al., 2019). Furthermore, HLRE has been shown to elicit greater neural drive compared to moderate resistance exercise when performed to failure under free‐flow conditions (Miller et al., 2020). Contextually, surface electromyography (sEMG) is commonly used to quantify skeletal muscle excitation during acute bouts of RE (Lacerda et al., 2019; Morton, Sonne, et al., 2019), while reductions in the post‐ compared to pre‐exercise maximum voluntary isometric contractions (MVICs) are used to quantify neuromuscular fatigue (Hill et al., 2022; Izquierdo et al., 2009; Karabulut et al., 2010).

Emerging evidence suggests that blood flow‐restricted RE (BFR‐RE) is an effective exercise modality for inducing skeletal muscle hypertrophy, even when performed using relatively low loads (e.g., 20%–30% 1‐RM, LLBFR) (Patterson et al., 2019). LLBFR may be advantageous in individuals where HLRE may be contraindicated (e.g., post‐anterior cruciate ligament reconstruction, post‐fracture rehabilitation, etc.) (Banwan Hasan & Awed, 2024; Ohta et al., 2003; Patterson et al., 2017, 2019). Clinically, LLBFR is gaining traction as a viable pre‐ and post‐surgical rehabilitation procedure in patients due to its ability to improve and maintain muscle mass while minimizing injury risk (Hughes et al., 2017; Ogawa et al., 2021). Mechanistically, LLBFR has been suggested to result in greater skeletal muscle excitation measured using sEMG than load‐matched free flow RE (Lacerda et al., 2019; Loenneke et al., 2015) while also achieving levels of skeletal muscle activation that are observed in response to free flow HLRE in some (Takarada et al., 2000) but not all studies (Biazon et al., 2019).

BFR‐RE‐induced increases in muscle hypertrophy have been hypothesized to be due to a combination of increased fiber recruitment, accumulation of metabolites, transient cellular swelling, and stimulation of muscle protein synthesis (Loenneke et al., 2012; Pearson & Hussain, 2015; Wilson et al., 2013), which are indicative of greater muscle excitation and enhanced cellular stress (Schoenfeld, 2013). A recent meta‐analysis that systematically reviewed low‐load resistance exercise with and without BFR (LLBFR and low‐load resistance exercise (LLRE), respectively) concluded that LLBFR had greater exercise‐induced muscle excitation than LLRE (Centner & Lauber, 2020). Although BFR‐RE has been shown to enhance skeletal muscle excitation in both trained and untrained adults, few studies have directly compared the acute impact of BFR‐RE on skeletal muscle excitation, total muscle activation, and neuromuscular fatigue in adults of different training statuses relative to traditional free‐flow HLRE. Therefore, the purpose of this study was to determine if there are differences in the muscle excitation and muscle activation of the vastus lateralis measured using sEMG in resistance‐trained (RT) versus untrained (UT) college‐aged males performing BFR‐RE at low loads (25% 1‐RM, LLBFR) or medium loads (50% 1‐RM, MLBFR) compared to a traditional free‐flow HLRE program (75% 1‐RM). Furthermore, we also sought to determine if there were differences in neuromuscular fatigue measured during a standardized isometric fatigue test in RT and UT college‐aged males following acute bouts of LLBFR, MLBFR, and HLRE. We hypothesized that the RT group would have higher absolute muscle excitation and lower relative muscle excitation during LLBFR, MLBFR, and HLRE than the UT group. We also hypothesized that the LLBFR and MLBFR would result in muscle excitation, muscle activation, and neuromuscular fatigue similar to HLRE despite lower training volumes.

## 2 METHODS

### 2.1 Ethics statement

The Louisiana State University's Institutional Review Board approved the study protocol and consent form for this study (IRBAM‐22‐0600), which was registered at clinicaltrials.gov (NCT05586451). All participants provided written and informed consent before their participation in the study, while all procedures conducted were in accordance to the Declaration of Helsinki.

### 2.2 Participants

Thirty‐two participants qualified for this study. Two participants were excluded due to noncompliance (i.e., only completed one study visit), and one participant was excluded due to technical (equipment) problems during their exercise visits. Thus, twenty‐nine healthy college‐aged males completed this study. The resistance‐trained participants (RT, *n* = 15) were required to report RE at least 3 days per week for 2 years. The untrained participants (UT, *n* = 14) exercised for less than 2 days per week and were required to report not performing RE training for at least 6 months before starting the study. All participants were free of any cardiovascular or metabolic diseases or other abnormalities preventing them from performing exercise. All participants were tobacco‐ and medication‐free, normotensive, and with no history of thromboembolism, sickle cell trait, or sickle cell anemia.

### 2.3 Study design

A randomized, repeated measures design was used to test the impact of acute HLRE, LLBFR, and MLBFR bouts on muscle excitation, muscle activation, and neuromuscular fatigue in RT and UT college‐aged males. The participants completed one screening visit, one strength training visit, and three acute exercise visits (HLRE, LLBFR, MLBFR). Research randomizer (https://randomizer.org/) was used to block randomize the three exercise conditions stratified by training status. The participants were not blinded to which exercise condition they were completing during a given trial.

### 2.4 Screening visit

The screening visit consisted of informed consent, medical history, and a physical activity readiness questionnaire for everyone (PARQ+) (Warburton, 2019), International Physical Activity Questionnaire (Sember et al., 2020), Muscle Strengthening Exercise Questionnaire (MSEQ) (Shakespear‐Druery et al., 2022), demographics, anthropometric measurements, and blood pressure. Height and body mass were measured using a stadiometer (Seca, Germany) and an electronic scale (Seca, Germany). Participants also completed a whole‐body Dual‐energy X‐ray Absorptiometry (DXA) scan (Horizon‐A, Hologic Inc., Danbury, CT, USA) as previously described (Davis et al., 2024; Wong et al., 2023). In addition, we quantified the thigh bone‐free lean mass of the dominant leg using region of interest (ROI) analyses described previously (Hirsch et al., 2021). They were also familiarized with the exercise equipment, testing protocol, and BFR.

### 2.5 Strength testing visit

All participants completed their strength testing visits at least 48 h after the screening visit and ~48 h or more after their last leg training session, if they were resistance‐trained, to avoid the potential confounding effect of muscle soreness on strength outcomes. All strength testing and exercise sessions were performed on the dominant leg, the leg the participant felt most comfortable kicking a ball. While participants were sitting on the isokinetic dynamometer (Biodex System 3, Shirley, NY), the total thigh length was measured from the greater trochanter to the lateral border of the patella's base. Surface EMG (sEMG) electrodes (Biopac Systems, Inc.™, Goleta, CA) were placed on the belly of the *vastus lateralis* at 1/3 the length of the thigh from the lateral border of the patella's base. The inter‐electrode distance was 20 mm, with the positive electrode superior to the negative electrode, and the ground electrode was placed on the patella according to the SENIAM guidelines (Hermens et al., 2000). Using the isotonic setting, we measured the knee extension one repetition max (1‐RM), which was determined as the maximum amount of torque that could be lifted through a full range of motion, quantified in Nm. We then measured the participants' peak isokinetic torque, quantified in Nm for the knee extension at 60^0^/s and range of motion set at 70^0^ (80–10^0^ where 0^0^ = full extension). Participants then performed a knee extension isometric endurance test with a joint angle set at 60^0^ of flexion (0^0^ = full extension). Participants performed an MVIC for 5 s, followed by a 5 s rest, and continued for 4 min (24 total MVICs). We adopted the isometric endurance test to reduce signal noise for sEMG readings (Armatas et al., 2010).

### 2.6 Exercise visits

**FIGURE 1:**
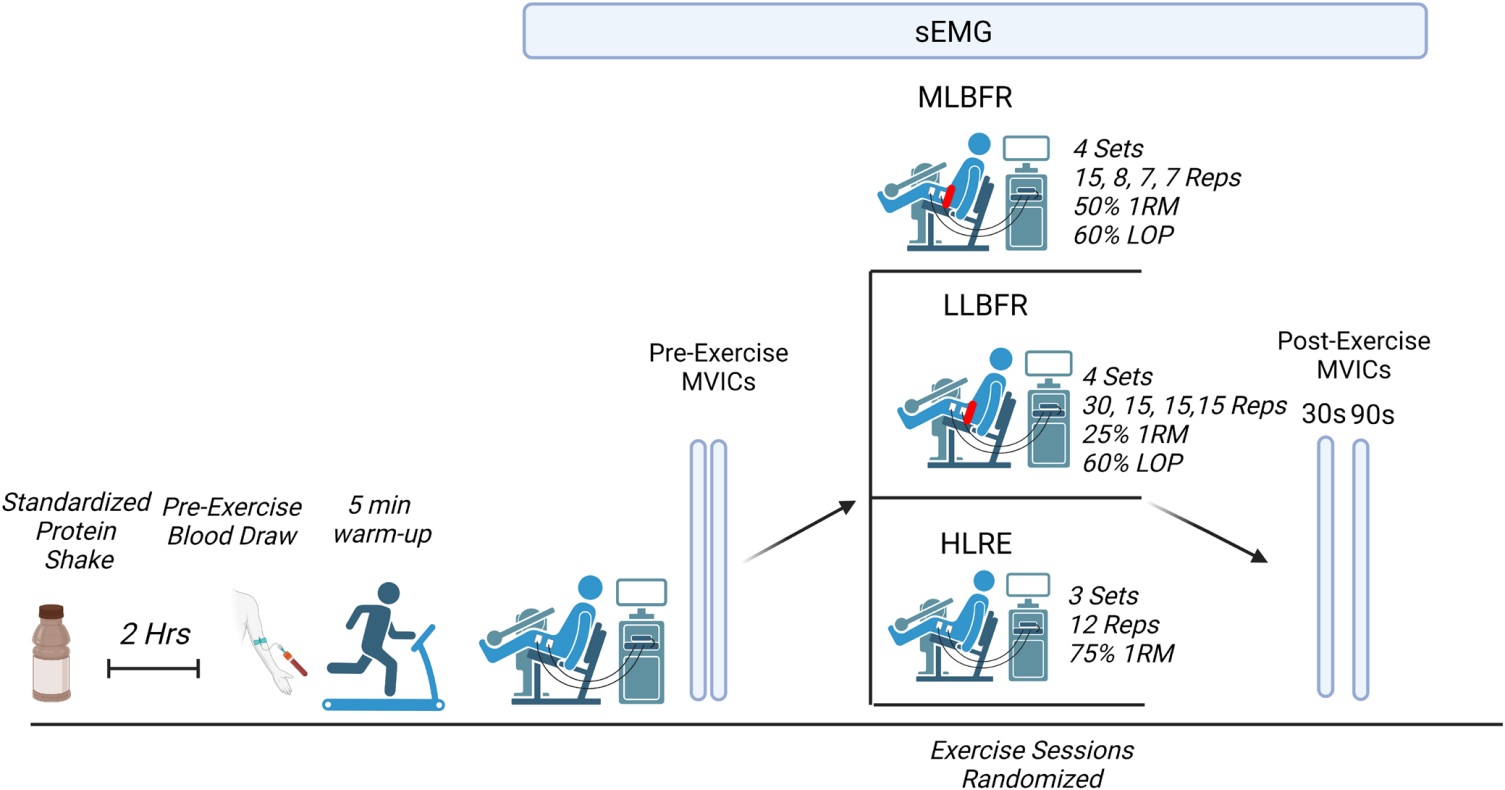
Overview of study flow during the single‐legged high load resistance exercise (HLRE), low load blood flow restricted resistance exercise (LLBFR), and moderate load blow flow restricted resistance exercise (MLBFR) study visits. Muscle excitation and total muscle activation were measured by surface electromyography (sEMG) on the vastus lateralis from their exercised leg. 1RM, 1 repetition maximum; LOP, limb occlusion pressure; MVIC, maximum isometric voluntary contraction; Reps, repetitions. This schematic was prepared in *BiorRender.* Irving, B. (2025) https://BioRender.com/n25e758.

All exercise visits were performed at least 48 h after the previous visit and at least 48 h after the last leg training day for trained participants to avoid the confounding effects of muscle soreness on the study outcomes. Figure 1 presents the overall study flow for each exercise visit. Participants were instructed not to have any food or beverages except water for at least 10 h before the start of their study days. Participants were provided a standardized breakfast (Boost™ Max, 30 g protein, 1 g sugar), which they were asked to consume 2 h before each exercise visit. The standardized meal was used to mimic a pre‐workout meal. All exercise visits were performed in the morning and, when possible, at the same time of day for each participant. Upon arrival and after a 5‐min rest period in a seated position, a pre‐exercise blood sample was obtained by venipuncture of an antecubital vein. Next, participants completed a 5‐min warm‐up on a treadmill at a self‐selected pace (≥1.5 mph) before being positioned on the Biodex. Participants were equipped with sEMG probes over the vastus lateralis of their dominant leg as described above (strength testing visit). The participants performed two pre‐exercise MVICs with a 1‐min break between pre‐exercise MVICs and then performed one of three randomly assigned exercise sessions (HLRE, LLBFR, and MLBFR). After completing the assigned exercise session, two MVICs were performed at ~30 s and ~90 s post‐exercise (1‐min break between postexercise MVICs), followed by a postexercise blood draw. The postexercise blood draws were taken ~3–5 min after completing the exercise session. Details of the HLRE, LLBFR, and MLBFR protocols are below.

### 2.7 Exercise protocols

1. High‐load resistance exercise (HLRE)—Participants performed three sets of isotonic knee extensions on the Biodex at 75% of 1‐RM for 12 repetitions with a 1‐min break between sets. One repetition (concentric and eccentric) was completed every 2 s to minimize sEMG signal noise.
2. Low‐load blood flow restricted resistance exercise (LLBFR)—For the LLBFR, we followed the consensus guidelines for BFR (Patterson et al., 2019). In brief, participants performed four sets of isotonic knee extensions on the Biodex at 25% of 1‐RM for 30, 15, 15, and 15 repetitions with a 1‐min break between sets, with one repetition every 2 s. The Delfi™ Personal Tourniquet System (PTS) and ~11.4 cm wide Easi‐Fit Tourniquets (Vancouver, CA) induced BFR. An Easi‐Fit Tourniquet was attached at the most proximal portion of the exercising thigh. Each participant's limb occlusion pressure (LOP) was determined using the PTS's built‐in Doppler system to measure and regulate the LOP at 60% throughout the entire exercise session. BFR was initiated immediately before the start of the first exercise set and terminated immediately following the completion of the last exercise set (~6–6.5 min of occlusion).
3. Medium‐load blood flow restricted exercise (MLBFR)—Participants performed four sets of isotonic knee extensions on the Biodex at 50% of 1‐RM for 15, 8, 7, and 7 repetitions with a 1‐min break between sets, with one repetition every 2 s. The BFR during the MLBFR was performed as described for the LLBFR (~4.5–5.25 min of occlusion). We chose this protocol to double the resistance exercise intensity while matching the training volume achieved during the LLBFR condition.

### 2.8 Blood draws and pre‐exercise plasma glucose

Following an overnight fast (≥10 h) and 2 h after a standardized protein shake (Boost™ Max, 30 g protein, 1 g) for breakfast, a pre‐exercise venous blood sample was collected from an antecubital vein into K_2_EDTA tubes (BD, Franklin Lakes, NJ). All blood draws were performed in a semi‐recumbent position. The whole blood was centrifuged at 500 *g* for 10 min at 4°C, and the plasma was stored at −80°C until analysis. Plasma blood glucose concentrations were assessed using the glucose oxidase method (Analox GL5 Analox Instruments, Lunenberg, MA).

### 2.9 Surface electromyography (sEMG) and signal processing

Participants were fitted with portable sEMG electrodes (Biopac Systems, BIONOMADIX, Goleta, CA) on their *vastus lateralis* to measure muscle excitation and total muscle activation as described above. The electrodes were connected to an amplifier and digitizer (Biopac Systems, EMG‐R2, Inc.™, Goleta, CA). The raw data were sampled at a rate of 2000 Hz and analyzed using AcqKnowledge 5.0 software (Biopac Systems, Inc.™, Goleta, CA). The bandwidth filter was set at 5 Hz–500 Hz, and the signal was amplified (gain: ×2000). The sEMG data were analyzed using a 30 ms moving window when performing the root mean square (RMS) analyses. Muscle activations were initially identified using the locate muscle activation function in BIOPAC's EMG analysis toolkit, which was followed by manual clean‐up to ensure that the RMS data were quantified from the onset and offset of each muscle action (e.g., MVIC or repetition). Thus, the EPOCHs for the MVICs were 5 s, and for the individual muscle repetition were ~2 s (inclusive of both the concentric and eccentric phases). Next, the maximal RMS amplitudes (AMP) per MVIC and per repetition were quantified in mV to determine muscle excitation. In addition, the integrated area under the EMG curve (iEMG) per MVIC and per repetition was quantified in mV∙s to determine total muscle activation. During the HLRE, LLBFR, and MLBFR, the maximal RMS AMP and iEMG for each complete repetition were quantified. The maximal RMS AMP measured for each repetition was normalized to the maximal RMS AMP measured during the pre‐exercise MVIC to quantify the relative muscle excitation. The iEMG measured per repetition was summed together (∑iEMG) to quantify the total muscle activation per exercise session.

### 2.10 Statistical analysis

**FIGURE 2:**
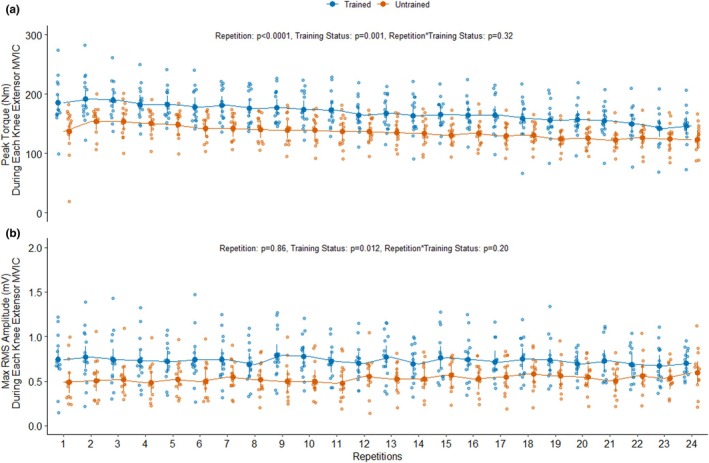
The peak torques measured for each maximal voluntary isometric contraction (MVIC) during 24 consecutive repetitions of the knee extensor endurance test are presented in panel (a) stratified by training status (Trained: *n* = 15, Untrained: *n* = 14). The maximal root means square (RMS) amplitudes measured for each MVIC during the knee extensor endurance test are presented in panel (b) stratified by training status (Trained: *n* = 14, Untrained: *n* = 13). One untrained participant had missing EMG data for the knee extensor endurance test. Data are LSMEANS ± 95% confidence intervals along with individual data points. The main effects of the training status, repetitions, and their interaction were assessed using mixed effects models.

**FIGURE 3:**
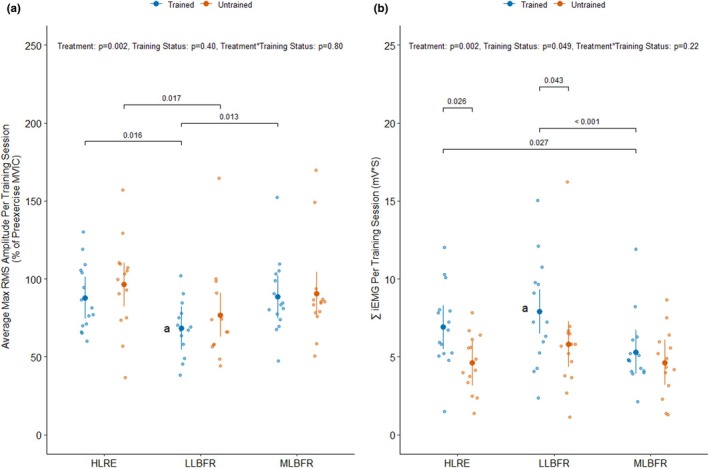
To quantify the relative muscle excitation, the max root means square (RMS) amplitude measured during each repetition was normalized to the max RMS amplitude measured during the pre‐exercise knee extensor maximal voluntary contraction (MVIC) and reported as a percentage. The mean relative muscle excitation across all repetitions within a given exercise treatment (HLRE, high load resistance exercise; LLBFR, low load blood flow restriction resistance exercise; MLBFR, medium load blood flow restriction resistance exercise) was used as the dependent variable for the data presented in panel (a) stratified by training status (Trained: *n* = 15, Untrained: *n* = 14). To quantify the total muscle activation, the integrated area under the curve of the electromyography RMS signal (iEMG) measured for each repetition was summed together (∑iEMG) within a given exercise treatment. The ∑iEMG was used as the dependent variable for the data presented in panel (b) stratified by training status. The main effects of the training status, treatment, and their interaction were assessed using mixed effects models. The *p* values above the brackets are for pairwise comparisons using “emmeans”. A: *n* = 14. One trained participant had missing EMG data for the LLBFR condition, while one trained participant was only able to complete the first two sets of the LLBFR treatment.

**FIGURE 4:**
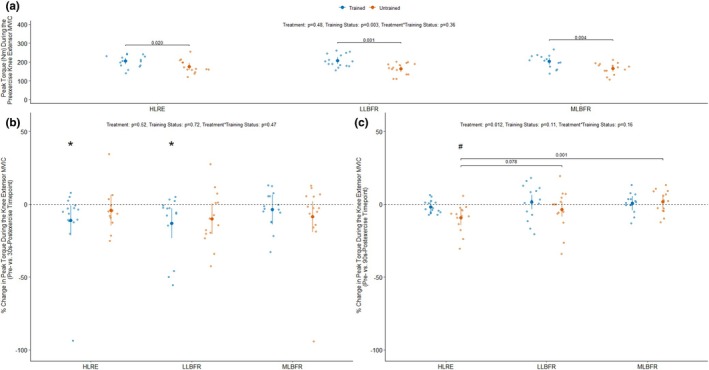
The peak torques measured during each pre‐exercise knee extensor maximal voluntary isometric contraction (MVIC) are presented in panel (a) stratified by training status (Trained: *n* = 15, Untrained: *n* = 14) and treatment (HLRE, high load resistance exercise; LLBFR, low load blood flow restriction resistance exercise; MLBFR, medium load blood flow restriction resistance exercise). The % change in peak torque measured during the knee extensor MVICs at the 30 s‐ and 90 s‐postexercise timepoint relative to pre‐exercise MVIC are presented in panels (b) and (c) stratified by training status and treatment. Data are LSMEANS ± 95% confidence intervals along with individual data points. The main effects of the training status, treatment, and their interaction were assessed using mixed effects models. *p < 0.05 and ^#^ *p* < 0.001 for a priori comparisons versus pre‐exercise (i.e., % change = 0) and the *p* values above the brackets are for pairwise comparisons using “emmeans”.

**FIGURE 5:**
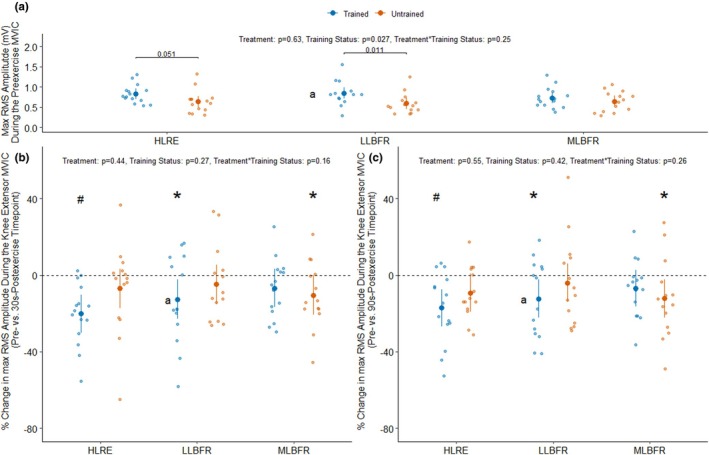
The maximal root means square (RMS) amplitudes measured during each pre‐exercise knee extensor maximal voluntary isometric contraction (MVIC) are presented in panel (a) stratified by training status (Trained: *n* = 14, Untrained: *n* = 13) and treatment (HLRE, high load resistance exercise; LLBFR, low load blood flow restriction resistance exercise; MLBFR, medium load blood flow restriction resistance exercise). The % change in maximal RMS amplitudes measured during the knee extensor MVICs at the 30 s‐ and 90 s‐postexercise timepoints relative to the pre‐exercise MVIC are presented in panels (b) and (c) stratified by training status and treatment. Data are LSMEANS ± 95% confidence intervals along with individual data points. The main effects of the training status, treatment, and their interaction were assessed using mixed effects models. *p < 0.05 and ^#^ *p* < 0.001 for a priori comparisons versus pre‐exercise (i.e., % change = 0) and the *p* values above the brackets are for pairwise comparisons using “emmeans”. A: *n* = 14. One trained participant had missing EMG data for the LLBFR condition, while one trained participant was only able to complete the first two sets of the LLBFR treatment.

Data were analyzed using Rstudio (2024.04.2 Build 764). Table 1 presents participant characteristics (mean ± SD) stratified by training status (Trained vs. Untrained) using the *tidyverse* (Wickham et al., 2019) and *gtsummary* (Sjoberg et al., 2021) packages. Differences between the trained and untrained were determined using Fisher's exact test for categorical variables and Welch's Two Sample *t*‐test for continuous variables using *gtsummary* (Sjoberg et al., 2021). Table 2 presents the exercise data (mean ± SD) stratified by training status (trained and untrained) and treatment (HRLE, LLBFR, and LLBFR) using the *tidyverse* (Wickham et al., 2019), *gtsummary* (Sjoberg et al., 2021), and *flextable* (Gohel & Skintzos, 2024) packages. Linear mixed‐effects models were used to detect differences between training status, treatments, and their interaction using restricted maximum likelihood (REML) (Bates et al., 2015; Kuznetsova et al., 2017). Specifically, the *lemr* function in the *nmle4* and *lmerTest* packages was used to fit the linear mixed‐effects models. In addition, ID was included in the linear mixed model as a random effect (lmer(y ~ training status + treatment + training status*treatment + 1|ID)). The Kenward‐Rogers method was used to determine the denominator degrees of freedom (Kenward & Roger, 1997). Data are presented as LSMEANS ± 95% confidence intervals. Post hoc linear contrasts were performed using the *emmeans* package and *pairs* function (Lenth, 2024). For the endurance test, the linear mixed models included the main effects of repetitions (REP1‐REP24), training status (trained and untrained), and their interaction. In addition, the participant ID was used as a random effect, as previously described. A similar model assessed differences in maximal RMS amplitudes measured during the muscle endurance test. Likewise, linear mixed‐effects models were fit for the primary study outcomes, where the main effects were training status, treatment, and their interaction, while the participant ID was used as a random effect. The primary outcomes were knee extensor peak torque and maximum RMS amplitude during the pre‐exercise MVICs, the percent change in peak torque, and maximum RMS amplitude measured during the postexercise MVIC relative to the pre‐exercise MVIC, and the relative and absolute amount of muscle activation that were achieved during the three exercise treatments. The maximal RMS amplitude measured for each repetition was normalized to the maximal RMS amplitude measured during the pre‐exercise MVIC to quantify the relative muscle activation and expressed as a percentage (%MVIC). The mean relative muscle activation across all repetitions within a given exercise treatment was used as the dependent variable. To quantify the total muscle activation, the iEMG measured for each repetition was summed across all repetitions ∑iEMG within a given exercise treatment. The ∑iEMG within a given exercise treatment was used as the dependent variable. The secondary outcomes included plasma glucose and cortisol measures before and after the exercise treatments. Figures 2, 3, 4, 5 were created using the *ggplot_the_response* function (Walker, 2024). For all statistical tests, an alpha level of <0.05 was used.

**TABLE 1:** Participant characteristics stratified by training status.

Characteristic	Trained, *N* = 15^a^	Untrained, *N* = 14^a^	*p* Value^b^
Race			
Asian	2/15 (13%)	1/14 (7.1%)	0.57
Black or African American	1/15 (6.7%)	1/14 (7.1%)	
Hispanic or Latino	0/15 (0%)	2/14 (14%)	
Mixed	2/15 (13%)	0/14 (0%)	
White	10/15 (67%)	10/14 (71%)	
Age (yrs)	20.6 (1.3)	20.6 (1.4)	0.96
Height (cm)	174.8 (6.5)	177.6 (5.7)	0.23
Weight (Kg)	78.0 (6.5)	76.7 (10.2)	0.69
BMI (Kg/m^2^)	25.6 (2.6)	24.4 (3.2)	0.27
Waist circumference (cm)	81.1 (4.6)	81.7 (8.9)	0.82
Whole‐body fat mass (%)	17.7 (3.9)	20.2 (4.1)	0.11
Whole‐body fat mass (Kg)	14.3 (3.9)	15.9 (4.8)	0.33
Whole‐body lean mass (Kg)	62.8 (4.6)	59.0 (6.5)	0.090
Thigh fat (%)	19.2 (4.5)	22.9 (5.0)	0.046
Thigh fat mass (Kg)	1.9 (0.5)	2.1 (0.7)	0.29
Thigh lean mass (Kg)	7.7 (0.8)	7.0 (1.0)	0.030
IPAQ‐vigorous (MET‐min/week)	1696.0 (986.4)	197.1 (351.0)	<0.001
IPAQ‐moderate (MET‐min/week)	1158.7 (1072.3)	145.7 (258.6)	0.003
IPAQ‐total (MET‐min/Week)	4532.3 (1791.9)	2275.7 (1825.1)	0.002
Resistance exercise (sessions/week)	4.4 (1.0)	0.2 (0.8)	<0.001
Resistance exercise (min/session)	100.7 (29.9)	10.4 (26.0)	<0.001
1‐RM (Nm)	81.0 (16.4)	74.6 (11.2)	0.23
Isokinetic 60°/s (Nm)	176.1 (36.3)	162.6 (39.1)	0.34

^a^ *n*/*N* (%); Mean (SD).

^b^ Fisher's exact test (Categorical Variables); Welch Two Sample *t*‐test (Continuous).

**TABLE 2:** Exercise data stratified by training status and treatment.

Characteristic	Resistance trained			Untrained			*p* ^b^	*p* ^b^	*p* ^b^
	HLRE	LLBFR	MLBFR	HLRE	LLBFR	MLBFR			
	*N* = 15^a^	*N* = 15^a^	*N* = 15^a^	*N* = 14^a^	*N* = 14^a^	*N* = 14^a^	Treatment	Training status	Treatment*training status
Total volume (Nm)	2194 (444)	1468 (207)^#^	1500 (308)^#^	2021 (301)	1414 (218)^#^	1390 (201)^#^	<0.001	0.28	0.29
Total concentric work (Joules)	3525 (583)	2771 (748)^#^	2611 (529)^#^	3296 (490)	2822 (641)^#^	2538 (425)^#,¥^	<0.001	0.66	0.28
Heart rate (bpm)	89 (9.3)	89 (10)	94 (8.3)	97 (9.9)	93 (14)	96 (7.4)	0.18	0.13	0.41
RPE (Borg 6–20)	13 (1.2)	13 (1.8)	13 (1.8)	12 (2.1)	13 (2.5)*	14 (1.3)*	0.010	0.90	0.24

Abbreviations: HLRE, high load resistance exercise; LLBFR, low load blood flow restricted resistance exercise; MLBFR, medium load blood flow restricted resistance exercise; RPE, ratings of perceived exertion.

^a^ Mean (SD).

^b^ *p* Values from linear mixed effects models.

*p < 0.05 (vs. HLRE within‐training status), ^#^ *p* < 0.001 (vs. HLRE within‐training status), and ^¥^ *p* < 0.05 (vs. LLRE within‐training status).

### 2.11 Sample size considerations

A sample size of at least *n* = 12 per group was selected based on sample sizes from prior studies (8–12 subjects per group) (Cook et al., 2013; Kubo et al., 2006; Sousa et al., 2017). Using sample size procedures outlined by Beck (2013), G*Power suggests that a sample size of 10 participants per group provides 80% power to detect an effect size of 1.0 at an *α* = 0.05 for detecting within‐participant differences in muscle activation based on paired data (e.g., LLBFR vs. MLBFR). Likewise, a sample size of 12 participants per group provided 80% power to detect an effect size of 1.2 at an *α* = 0.05 for between‐participant differences in muscle excitation based on independent data (e.g., RT vs. UT). Although these effect sizes are often considered large, pre‐ to post‐training effect sizes for muscle excitation and changes in strength have been reported to be greater than 1.0 following only 6 weeks of HLRE and LLBFR (Sousa et al., 2017).

## 3 RESULTS

### 3.1 Participants characteristics

The overall participant characteristics stratified by training status are presented in Table 1. The RT participants reported 8.6 times more vigorous (*p* < 0.001), 8 times more moderate (*p* = 0.003), and 2 times more total MET‐minutes per week of physical activity (*p* = 0.002). By design, the RT participants reported a greater number of resistance exercise sessions (*p* < 0.001) and minutes per training session (*p* < 0.001) than the UT participants, as estimated by the MSEQ. However, neither the knee extension 1‐RM nor the peak isokinetic torque measures differed between the RT and UT groups (*p* = 0.23 and *p* = 0.34, respectively). The RT participants had 10% more thigh lean mass than the UT participants (*p* = 0.03). The pre‐exercise plasma glucose concentrations were not different between treatments, training status, or their interaction (all *p* > 0.05). The pre‐exercise plasma glucose concentrations were 5.6 mM (5.4–5.9 mM) in the RT and 5.7 mM (5.4–5.9 mM) in the UT participants, averaged over all treatment levels.

### 3.2 Isometric knee extensor endurance test

During the knee extensor endurance test, the RT participants produced 24% higher average peak torque than the UT participants (*p*_Training Status_ = 0.001, Figure 2a). Peak torque per repetition declined throughout the endurance test (*p*_Repetition_ < 0.0001, Figure 2a) independent of training status (*p*_Repetition*Training Status_ = 0.32, Figure 2a). Likewise, both groups had similar reductions in peak torque when comparing the peak torque achieved during the first 4 MVICs with the last 4 MVICs (−18 ± 19% vs. −17.3 ± 9%, *p* = 0.89 Welch's Two Sample *t*‐test). The RT participants produced 38% higher absolute maximal RMS AMP (mV) during the knee extensor endurance test than the UT participants (*p*_Training Status_ = 0.012, Figure 2b). However, the absolute maximal RMS AMP per repetition did not change throughout the endurance test (*p*_Repetition_ = 0.86 and *p*_Repetition*Training Status_ = 0.20, Figure 2b).

### 3.3 Exercise session data

The total volume (load*repetitions or Nm*repetitions) was higher during the HLRE treatment compared to both the LLBFR (46% higher, *p*_between‐treatments_ <0.001) and HLBFR (46% higher, *p*_between‐treatments_ < 0.001) treatments, independent of training status (*p*_Training Status_ = 0.28 and *p*_Training Status*Treatment_ = 0.29) (Table 2). By design, the total volume was not different between the LLBFR and MLBFR treatments (*p* > 0.05). The total concentric work measured during the HLRE treatment was higher than the LLBFR (22% higher, *p*_between‐treatments_ < 0.001) and MLBFR (32% higher, *p*_between‐treatments_ < 0.001) treatments, independent of training status (*p*_Training Status_ = 0.66 and *p*_Treatment*Training Status_ = 0.28) (Table 2). The total concentric work was not different between the LLBFR and MLBFR treatments among the RT participants (*p* > 0.05) (Table 2). However, the total concentric work was 11% higher during LLBFR than MLBFR (*p*_between‐treatments_ = 0.026) within the UT participants (Table 2). The overall correlation between the total volume versus total concentric work was high (*r* = 0.82, *p* < 0.001). One trained participant could only complete the first two sets during the LLBFR treatment. The average heart rates during each exercise session were not different between treatments, independent of training status (all *p* > 0.05) (Table 2). However, the RPEs were higher in response to the LLBFR and MLBFR treatments than the HLRE treatments within the UT participants (*p*_between‐treatments_ < 0.05) (Table 2).

### 3.4 Muscle excitation measured during the exercise sessions

The mixed effects models revealed that there were differences in the normalized RMS AMP (%MVIC) per training session between three exercise treatments (*p*_Treatment_ = 0.002), independent of training status (*p*_Training Status_ = 0.40 and *p*_Treatment*Training Status_ = 0.80) (Figure 3a) indicative of differences in relative muscle excitation. Specifically, the RMS AMP (%MVIC) was 26.7% higher during the HLRE than the LLBFR sessions (*p*_between‐treatments_ = 0.0009, averaged across all levels of training status) and 23.2% higher during the MLBFR than the LLBFR sessions (*p*_between‐treatments_ = 0.004, averaged across all levels of training status) (Figure 3a). Moreover, the RMS AMP (%MVIC) was 28.2% higher during the HLRE than the LLBFR sessions (*p*_within‐training status_ = 0.016) and 29.1% higher during the MLBFR than the LLBFR sessions within the RT participants (*p*_within‐training status_ = 0.013) (Figure 3a). In addition, the RMS AMP (%MVIC) was 25.4% higher during the HLRE than the LLBFR sessions within the UT participants (*p*_within‐training status_ = 0.017) (Figure 3a).

### 3.5 Total muscle activation measured during the exercise sessions

The mixed effects models revealed that there were differences in ∑iEMG (mV∙s) per training session between three exercise treatments (*p*_Treatment_ = 0.002) and training status (*p*_Training Status_ = 0.049), but not their interaction (*p*_Treatment*Training Status_ = 0.22) (Figure 3b) indicative of differences in total muscle activation. Specifically, the ∑iEMG was 33.9% higher in the RT than in the UT participants (*p*_Training Status_ = 0.049, averaged over the levels of treatment) (Figure 3b). The ∑iEMG was 50.0% higher in RT than in the UT participants during the HLRE (*p*_between‐training status_ = 0.026) and 36.2% higher in the RT than in the UT participants during the LLBFR treatment (*p*_between‐training status_ = 0.043) (Figure 3b). The ∑iEMG was also 19.3% higher during LLBFR than during the HLRE treatment (*p*_between‐treatments_ = 0.034, averaged across all levels of training status) and 38.1% higher during the LLBFR than during the MLBFR treatment (*p*_between‐treatments_ = 0.0005, averaged across all levels of training status) (Figure 3b). Moreover, the ∑iEMG was 30.0% higher in HLRE than in the MLBFR treatments (*p*_within‐training status_ = 0.027) and 49.1% in LLBFR than the MLBFR treatments (*p*_within‐training status_ < 0.001, Figure 3b) in the RT participants (Figure 3b).

### 3.6 Peak torque measured during the pre‐ and post‐exercise knee extensor MVICs

The RT participants produced 21% higher peak torque measured during the pre‐exercise MVICs than the untrained participants (*p*_Training Status_ = 0.003, averaged across all levels of treatment) (Figure 4a). Notably, no differences in peak torque were measured during the pre‐exercise MVICs between treatments (*p*_Treatment_ = 0.48) (Figure 4a). There were no differences in the percent change (%Δ) in peak torque measured during the postexercise MVIC relative to the pre‐exercise MVIC between treatments (*p*_Treatment_ = 0.52), training status (*p*_Training Status_ = 0.72), or their interaction (*p*_Treatment*Training Status_ = 0.47) at the 30‐s postexercise timepoint (Figure 4b). However, the peak torque was −7.6% (*p*_within‐treatment_ = 0.043) and −11.6% (*p*_within‐treatment_ = 0.002) lower during the 30‐s postexercise MVIC than the pre‐exercise MVIC following the HLRE and LLBRF conditions, respectively, when averaged over all levels of training status (Figure 4b).

Moreover, the RT participants produced lower peak torque following the HLRE (−11.1%, *p*_within‐treatment_ = 0.034) and LLBFR (−13.2%, *p*_within‐treatment_ = 0.012) treatments during the 30‐s postexercise MVIC compared to the pre‐exercise MVIC (Figure 4b). In contrast, the UT participants did not show significantly lower peak torque during the 30‐s postexercise MVIC than the pre‐exercise MVIC regardless of treatment (Figure 4b). Figure 4c suggests that there were differences in the %Δ in peak torque measured during the 90‐s postexercise MVIC relative to the pre‐exercise MVIC between treatments (*p*_Treatment_ = 0.012), but not between training status (*p*_Training Status_ = 0.72) or their interaction (*p*_Treatment*Training Status_ = 0.16). Specifically, the peak torque measured during the 90‐s postexercise MVIC was lower than the pre‐exercise MVIC following HLRE treatment (−5.6%, *p*_within‐treatment_ = 0.002) when averaged over all levels of training status (Figure 4c). Moreover, the UT participants showed a lower peak torque during the 90‐s postexercise MVIC than the pre‐exercise MVIC following HLRE treatment (−9.4%, *p*_within‐treatment_ = 0.0004) (Figure 4c). Moreover, the %Δ in peak torque following HLRE treatment was greater than the MLBFR treatment (*p*_between_ = 0.001) within the UT participants (Figure 4c).

### 3.7 Muscle excitation during the pre‐ and post‐exercise knee extensor MVICs

The RT participants had a 28.6% higher maximal RMS AMP (mV) during the pre‐exercise MVICs than the UT participants (*p*_Training Status_ = 0.027, averaged across all levels of treatment) (Figure 5a), indicative of greater absolute muscle excitation. Notably, there were no differences in the maximal RMS AMP measured during the pre‐exercise MVICs between treatments (*p*_Treatment_ = 0.63) nor their interaction (*p*_Treatment*Training Status_ = 0.25) (Figure 5a). Figure 5b suggests that there are no differences in the %Δ RMS AMP between treatments (*p*_Treatment_ = 0.44), training status (*p*_Training Status_ = 0.27), or their interaction (*p*_Treatment*Training Status_ = 0.16) during the 30‐s postexercise MVIC relative to the pre‐exercise MVIC. The maximal RMS AMP measured during the 30‐s postexercise MVICs was lower than the pre‐exercise MVIC following HLRE (−13.6%, *p*_within‐treatment_ = 0.0003), LLBFR (−8.7%, *p*_within‐treatment_ = 0.019), and MLBFR (−8.7%, *p*_within‐treatment_ = 0.019) when averaged over all levels of training status (Figure 5a). However, the RT participants showed lower maximal RMS AMP following the HLRE (−20.2%, *p*_within‐treatment_ = 0.0001) and LLBFR (−12.6%, *p*_within‐treatment_ = 0.017) treatments during the 30‐s postexercise MVIC than the pre‐exercise MVIC (Figure 5b). The UT participants showed a lower maximal RMS AMP following LLBFR (−10.5%, *p*_within‐treatment_ = 0.047) during the 30‐s postexercise MVIC than the pre‐exercise MVIC (Figure 5b). Figure 5c suggests that there are no differences in the %Δ RMS AMP between treatments (*p*_Treatment_ = 0.55), training status (*p*_Training Status_ = 0.42), or their interaction (*p*_Treatment*Training Status_ = 0.26) during the 90‐s postexercise MVIC than the pre‐exercise MVIC. The maximal RMS AMP measured during the 90‐s postexercise MVICs was lower following HLRE (−13.2%, *p*_within‐treatment_ = 0.0003), LLBFR (−8.2%, *p*_within‐treatment_ = 0.023), and MLBFR (−9.6%, *p*_within‐treatment_ = 0.019) treatments when averaged over all levels of training status (Figure 5c). However, the RT participants showed lower maximal RMS AMP following the HLRE (−17.1%, *p*_within‐treatment_ = 0.0006) and LLBFR (−12.4%, *p*_within‐treatment_ = 0.016) treatments during the 90‐s postexercise MVIC than the pre‐exercise MVIC (Figure 5c). The UT participants showed a decline in maximal RMS AMP following LLBFR (−12.3%, *p*_within‐treatment_ = 0.016) treatments during the 90‐s postexercise MVIC than the pre‐exercise MVIC (Figure 5c).

### 3.8 Testing for the potential confounding effect of muscle size on muscle excitation

Since muscle size could be a confounding variable related to greater maximal RMS AMP in the RT group compared to the UT group (Skarabot et al., 2021), and prior work has normalized RMS AMP data by muscle cross‐sectional area (Keller et al., 2021), we performed exploratory analyses using the thigh lean mass as a covariate (Karp et al., 2012; Tanner, 1949). However, the addition of thigh lean mass as a covariate to the RMS AMP models neither changed their main effects nor their interactions and did not reach the significance level (all *p* > 0.05); these additional analyses were excluded from the present study.

## 4 DISCUSSION

We sought to determine differences in muscle excitation and total muscle activation of the vastus lateralis in resistance‐trained (RT) versus untrained (UT) college‐aged males performing acute bouts of LLBFR, MLBFR, and HLRE. Moreover, we examined whether there were differences in neuromuscular fatigue following the acute bouts of exercise. The present results suggest that the (relative) normalized muscle excitation (RMS AMP, %MVIC) measured during the HLRE and MLBFR treatments were higher than the LLBFR treatment, independent of the training status. As expected, the total muscle activation (∑iEMG) in response to the three exercise treatments was higher in the RT compared to the UT participants. The total muscle activation was also higher during the LLBFR treatment than the MLBFR and HLRE treatments. These data suggest that (i) training status had minimal impact on (relative) normalized muscle excitation in response to the three treatments, (ii) the RT participants achieved greater total muscle activation during the acute bouts of exercise, and (iii) despite lower (relative) normalized muscle excitation, LLBFR led to greater total muscle activation compared to the volume‐matched MLBFR and the HLRE treatments. Our results also show that the RT participants had lower peak torque measured during the ~30‐s postexercise MVIC following both HLRE and LLBFR, which returned to pre‐exercise levels (no change) by the 90‐s postexercise MVIC. In contrast, the UT participants only showed a lower peak torque during the ~90‐s postexercise MVIC than their pre‐exercise MVIC following HLRE. Our results also suggest that less muscle excitation was measured during the 30‐s and 90‐s postexercise MVIC following the HLRE and LLBFR among the RT participants. At the same time, muscle excitation was lower in the 30‐s and 90‐s postexercise MVIC than in the MLBFR among UT participants. These data suggest that reductions in muscle excitation may contribute to some of the exercise‐induced reductions in peak torque measured during an isometric knee extension MVIC.

Although the mechanisms of resistance training‐induced muscle hypertrophy are complex and remain to be fully elucidated, the degree of muscle excitation and total muscle activation achieved during training remains a potential factor activating some of the molecular transducers of muscle hypertrophy (Schoenfeld, 2013). Moreover, some have suggested that blood flow restriction can enhance relative muscle excitation induced by LLBFR (Hill et al., 2023; Loenneke et al., 2015), which could translate into greater muscle hypertrophy in LLBFR relative to LLRE (Davis et al., 2024). Consistent with our findings, another recent study showed that HLRE resulted in greater voluntary muscle excitation than LLBFR in untrained adults (Biazon et al., 2019); however, the exercise volume was higher in the HLRE condition compared to the LLBFR condition in both studies. Moreover, their study also showed that the HLBFR condition resulted in higher voluntary muscle excitation than the LLBFR condition (Biazon et al., 2019), which is consistent with our findings. Of note, our MLBFR condition was performed at 50% 1‐RM and was matched for volume to the LLBFR condition, while the HLRE was performed at 80% of 1‐RM and was a higher training volume than the LLBFR condition in the later study (Biazon et al., 2019). One could also argue that muscle hypertrophy may be more related to total neural drive (i.e., effort) than simple measures of muscle excitation and/or total muscle activation determined by sEMG (Morton, Colenso‐Semple, et al., 2019). Along these lines, recent data suggest that individuals seeking greater neural drive should perform HLRE rather than moderate LLRE (Miller et al., 2020). Unfortunately, measurements of total neural drive were beyond the scope of the present study. Thus, future studies are needed to measure the impact of HLRE, LLBFR, and MLBFR on total neural drive using methods outlined in Farina et al. (Farina et al., 2010).

Exercise‐induced neuromuscular fatigue is often characterized by lower knee extensor peak torque measured during a postexercise MVIC compared to the pre‐exercise MVIC (Hill et al., 2022). Our data suggest that HLRE, LLBFR, and MLBR lead to lower peak torque at ~30‐s postexercise MVIC than the pre‐exercise MVIC averaged across all levels of training status. In the present study, our RT participants returned to their pre‐exercise peak torque by the 90‐s postexercise timepoint. These findings are consistent with previous data that have suggested that BFR‐RE causes transient reductions in force production that are quickly resolved (Husmann et al., 2018; Loenneke et al., 2013). Consistent with the present study, Loenneke et al. (2013) showed similar results; BFR‐RE significantly reduced peak torque immediately postexercise but rebounded within an hour of recovery in RT adults (Loenneke et al., 2013). The studies by Loenneke and Husmann performed LLBFR at 30% 1‐RM with the traditional 30, 15, 15, and 15 repetitions. Our participants performed LLBFR at 25% 1‐RM, which may have been one of the contributing factors for our participants reaching baseline levels more quickly. Moreover, based on the pre‐exercise MVICs peak torques reported in the studies above (>250 Nm), their RT participants appear to be stronger than our RT participants (205 Nm). A recent study also demonstrated that LLRE and LLBFR performed at 20% 1‐RM lead to immediate reductions in maximal voluntary contraction force production in moderately RT adults (Pignanelli et al., 2024). Although the force production quickly rebounded, it remained lower than the baseline for up to 4 h.

Our UT participants showed no reduction in knee extensor MVIC peak torque at 30‐s postexercise but experienced a significant decline at 90 s in the HLRE treatment. However, there was a significant reduction in their knee extensor MVIC peak torque at 90‐s postexercise in the HLRE treatment. While our findings align with some studies (Fatela et al., 2016), they differ from others (Hill et al., 2022), possibly due to variability in MVIC measurements and study power. For example, Fatela et al. (2016) examined the impact of BFR‐RE at 20% 1‐RM and under a range of occlusion pressures (40%, 60%, and 80% LOP) in healthy UT adults. The primary finding of their study was that although there was an immediate reduction in knee extensor MVIC peak torque following the 80% LOP treatment, they did not observe reductions in MVIC peak torque following the 60% LOP treatment, which is comparable to the LOP implemented in the present study. In contrast, recent data suggest that both LLRE and LLBFR are sufficient to reduce the knee extensor MVIC peak torque using a protocol similar to ours (1 × 30, 3 × 15 repetition protocol) in recreationally active adults with no differences between treatments (Hill et al., 2022). Although it is not readily apparent why our UT participants did not show significant declines in their knee extensor MVIC peak torques following LLBFR, it may be due to insufficient power and slightly higher than expected variability in the MVIC peak torque measurements following the LLBFR condition. However, our untrained UT participants showed reductions in their knee extensor MVIC peak torque measurements at the 90‐s postexercise timepoint, which was significantly different from the LLBFR and MLBFR treatments.

Our data suggest that HLRE, LLBFR, and MLBR lead to reductions in the voluntary muscle excitation measured during the ~30 and ~90‐s postexercise MVICs relative to the pre‐exercise MVIC when averaged across all levels of training status, which is consistent with prior studies. Our RT participants had significant reductions in muscle excitation measured during the postexercise knee extensor MVICs relative to the pre‐exercise MVIC following the HLRE and LLBFR conditions at 30‐s and 90‐s timepoints. Our results are consistent with Husmann et al. (2018), who demonstrated significant reductions in voluntary muscle excitation measured during the immediate postexercise knee extensor MVIC relative to the pre‐exercise MVIC, which remained depressed until the 8‐min timepoint (Husmann et al., 2018). Moreover, Hill et al. (2022) also showed that LLBFR reduces voluntary muscle excitation measured during the immediate postexercise MVIC relative to the pre‐exercise MVIC in UT adults. Likewise, recent data also suggest that LLRE and LLBFR, when performed to failure, also result in reductions in voluntary muscle excitation during postexercise knee extensor MVICs relative to pre‐exercise MVICs in moderately trained adults (Pignanelli et al., 2024). In contrast, our UT participants did not have reductions in voluntary muscle excitation measured during their postexercise MVICs relative to the pre‐exercise MVIC following the HLRE or LLBFR conditions. Consistent with our findings, Fatela et al. (2016) also showed no reduction in voluntary muscle excitation measured during the postexercise MVIC following LLBFR compared to the pre‐exercise MVIC when performed at 40% or 60% LOP (Fatela et al., 2016). However, the voluntary muscle excitation measured during the ~30 and ~90 s postexercise MVICs were lower than the pre‐exercise MVIC following MLBFR in our UT participants. The reason for the reduction in voluntary muscle excitation following the MLBFR condition in our UT participants remains to be determined.

### 4.1 Strengths and limitations

One strength of the present study design is the within‐and between‐subject design. Another strength of the present study is that we used the Delfi PTS' patented LOP detection technology to measure and maintain the 60% LOP precisely during the two BFR conditions based on Doppler blood flow measurements, which trained Doppler ultrasound technicians have previously validated (Masri et al., 2016). Moreover, the Delfi PTS technology is designed to apply consistent pressure throughout an exercise session (Hughes et al., 2018). Our study has a few notable limitations. Although our RT participants had higher thigh bone‐free lean mass, pre‐exercise MVICs, and muscle endurance compared to the untrained participants, their isotonic 1‐RMs were not significantly different, which could have limited our ability to observe differences in muscle excitation, total muscle activation, and neuromuscular fatigue between RT and UT participants. Although the total muscle activation during the HLRE and LLBFR appears to be qualitatively higher in the RT than in the UT participants, these differences did not reach statistical significance. We suspect these differences may have reached the level of statistical significance if the difference in isotonic 1‐RMs had been greater and/or if we had more participants per group. Moreover, considering that our participants were healthy, college‐aged males, there may have been a few UT participants who were either naturally strong and/or under‐reported their resistance training history, further attenuating between‐training status differences in 1‐RM. Another potential limitation of the present study is that we did not measure muscle excitation during an MVIC plus maximum potentiated singlets or a peak twitch torque contraction to quantify voluntary muscle excitation more directly (Hill et al., 2022). Another limitation of this study was that it only included males. Future studies need to explore the impact of HLRE, LLBFR, and MLBFR on neuromuscular fatigue, muscle excitation, muscle activation, and sex‐based (male vs. female) differences in these parameters.

## 5 CONCLUSION

Our study showed that our resistance‐trained participants had greater absolute muscle activation than untrained colleged‐aged males during their knee extensor MVIC. However, muscle excitation was significantly lower postexercise for all three acute exercise conditions, independent of training status. Although LLBFR resulted in lower relative muscle excitation than the HLRE or MLBFR during treatments, the total muscle activation during the LLBFR was higher than both the HLRE and MLBFR treatments. This finding suggests that the greater number of repetitions combined with BFR may also be an essential driver of total muscle activation. Future studies should focus on the effects of the same training conditions used in this study on muscular fatigue, strength, excitation, and hypertrophy after a training intervention instead of an acute bout of exercise.

## AUTHOR CONTRIBUTIONS

BD, GS, NJ, TA, and BI developed the study design; BD, GS, and NJ collected data; BD, VF, and BI analyzed the data; BD, GS, NJ, TA, and BI wrote and edited the manuscript. All authors have read and agreed to the published version of the manuscript.

## CONFLICT OF INTEREST STATEMENT

All authors report no conflicts of interest. Delfi, Inc. provided their Personalized Tourniquet System used in the present study. However, Delfi, Inc. had no input in this manuscript's data analysis, interpretation, or writing of the present manuscript.

## Data Availability

Data are available upon request.
